# Strategies for Overcoming T-Wave Oversensing

**DOI:** 10.1016/s0972-6292(16)30823-3

**Published:** 2014-12-15

**Authors:** Serkan Cay, Ozcan Ozeke, Firat Ozcan

**Affiliations:** Department of Cardiology, Division of Arrhythmia and Electrophysiology, Yuksek Ihtisas Heart-Education and Research Hospital, Ankara, Turkey

**Keywords:** T-Wave Oversensing

We have read with great interest the article entitled 'Left and Right Ventricle Leads Switch as a Solution for T Wave Oversensing - How a Good Idea Turned out Bad' by Alzand et al. in the last issue of the journal [1]. They have tried to solve the problem of T-wave oversensing (TWOS) by switching ventricular leads from their responsible ports.

Atrial tachycardia/atrial fibrillation accounted for the majority of cardiac resynchronization therapy loss and inappropriate therapies. However, many programmable causes of cardiac resynchronization therapy loss and inappropriate therapies such as TWOS might also be detected. We should mention about potential solutions to overcome TWOS problem. Initially, programmable features of the device should be performed before the opening of the pace pocket due to various complications including device-related infection. As the authors stated that the right ventricular lead sensitivity can be decreased to acceptable values. Extremely decreasing sensitivity (>0.6 mV) is, however, already not a solution because of undersensing of ventricular tachyarrhythmia. Some devices have special programs including programmable threshold start, decay delay, upper threshold, T-wave blank period, high-pass filtering, differentiating waveform characteristics and frequencies of R and T waves, and ventricular tachycardia discriminators including morphology to suppress TWOS. Changing ventricular sensing configuration can result in higher R-wave amplitude (>3 mV) and loss of T-wave sensing. Shortening of the post-ventricular atrial refractory period is another option to overcome TWOS. However, this carries the risk of inappropriate therapies. Changing timing and sequence of ventricular pacing with various V-V pace delay settings can also solve the problem of TWOS. Because with changing sequence and timing of V-V pace ventricular repolarization preceding depolarization and T-wave morphology change. The last resort can be increasing therapy zones to high ranges and detection intervals to prevent delivery of inappropriate therapies although it seems inappropriate due to not delivery of programmed therapies to real tachyarrhythmia. If programmed parameters do not work invasive options including repositioning of the lead or implantation of a new lead with good sensing and pacing values should be performed. Therefore, the authors should have tried to perform some of above mentioned programmable parameters to overcome TWOS before implanting a new lead although the R-wave amplitude of the defibrillator lead was 1.5 mV. Second, the authors should have tried to perform repositioning of the defibrillator lead before implanting a new lead although it seems as a more complex procedure. Lastly, the locations of figures 1 and 3 seem wrong.

## References

[R1] Alzand BSN (2014). Left and Right Ventricle Leads Switch as a Solution for T Wave Oversensing - How a Good Idea Turned out Bad. Indian Pacing Electrophysiol J.

